# Will prenatal exposure to SARS-CoV-2 define a birth cohort with accelerated aging in the century ahead?

**DOI:** 10.1017/S204017442000104X

**Published:** 2020-11-10

**Authors:** Molly Crimmins Easterlin, Eileen M. Crimmins, Caleb E. Finch

**Affiliations:** 1 Keck School of Medicine of University of Southern California, LAC+USC Medical Center; 2 Leonard Davis School of Gerontology, University of Southern California; 3 Dornsife College, University of Southern California

**Keywords:** COVID-19, 1918 epidemic, exposure *in utero*

## Abstract

The 1918 Influenza pandemic had long-term impacts on the cohort exposed *in utero* which experienced earlier adult mortality, and more diabetes, ischemic heart disease, and depression after age 50. It is possible that the Coronavirus Disease 2019 (COVID-19) pandemic will also have long-term impacts on the cohort that was *in utero* during the pandemic, from exposure to maternal infection and/or the stress of the pandemic environment. We discuss how COVID-19 disease during pregnancy may affect fetal and postnatal development with adverse impacts on health and aging. Severe maternal infections are associated with an exaggerated inflammatory response, thromboembolic events, and placental vascular malperfusion. We also discuss how *in utero* exposure to the stress of the pandemic, without maternal infection, may impact health and aging. Several recently initiated birth cohort studies are tracking neonatal health following *in utero* severe acute respiratory syndrome virus 2 (SARS-CoV-2) exposure. We suggest these cohort studies develop plans for longer-term observations of physical, behavioral, and cognitive functions that are markers for accelerated aging, as well as methods to disentangle the effects of maternal infection from stresses of the pandemic environment. *In utero* exposure to COVID-19 disease could cause developmental difficulties and accelerated aging in the century ahead. This brief review summarizes elements of the developmental origins of health, disease, and ageing and discusses how the COVID-19 pandemic might exacerbate such effects. We conclude with a call for research on the long-term consequences of *in utero* exposure to maternal infection with COVID-19 and stresses of the pandemic environment.

## Introduction

By the end of 2020, about 300,000 infants may be born in the United States to mothers infected by the severe acute respiratory syndrome virus 2 (SARS-CoV-2) sometime during their pregnancy. Millions more will be born to mothers and families who have experienced tremendous stress and change in their daily lives and environments due to the pandemic. Postnatal effects of *in utero* exposure to SARS-CoV-2 from pregnant women with Coronavirus Disease 2019 (COVID-19) are mostly unknown, but are suggested from the 1918 Influenza pandemic which had lifelong effects on health and achievement. We discuss how maternal infections and *in utero* exposure to the pandemic environment may affect postnatal development and lifetime cardiovascular, metabolic, and neurodevelopmental health. The effects of SARS-CoV-2 on pregnant women and neonates are compared to other coronaviruses, SARS (severe acute respiratory syndrome) and MERS-CoV (Middle East Respiratory Syndrome). We then suggest indicators of maternal, neonatal, and later life health that should be monitored following *in utero* exposure to SARS-CoV-2. Lastly, we consider how to disentangle the effects of maternal infection versus the effects of prenatal exposure to the stress of the pandemic environment, both positive and negative.

### Lifetime effects of the 1918–1919 influenza pandemic

The 1918 Influenza pandemic (Influenza A, H1N1 subtype) had multiple health consequences for the cohort that was *in utero* during the pandemic.^1^ ^,^ ^2^ The U.S. cohort *in utero* during the peak pandemic period, experienced more depression after age 50, diabetes and ischemic heart disease after age 60, and mortality after age 65, with some variation in estimated effects.^2^ ^–^ ^5^ The Taiwanese cohort exposed *in utero* in 1919 had more renal disease, circulatory and respiratory morbidities, and diabetes after age 60.^6^ The corresponding Swedish cohort had higher morbidity at ages 54–87, indicated by excess hospitalization and male mortality from heart disease and cancer.^7^ The effect on late life health varied by trimester of development at the pandemic peak. Elevated heart disease in the U.S. was associated with second trimester exposure at the peak for the 1918–1919 cohort; while diabetes was associated with third trimester exposure at the peak.

No data on birth weight are available for the 1918 Influenza cohort; however, there are many indications that early life physical health and neurodevelopment were directly affected either by *in utero* exposure or indirectly by its multifarious stresses. Men in the 1918–1919 U.S. flu cohort were shorter at WWII enlistment than flanking cohorts,^2^ while children and adolescents in Taiwan were shorter than surrounding cohorts.^6^ U.S. women exposed *in utero* married men with less education.^3^ Educational deficits in the U.S. and Taiwan for these cohorts could be related to early cognitive deficits.^1^ ^,^ ^6^ Lower socioeconomic status (SES) as adults for those *in utero* during the 1918 epidemic was also confirmed in the parents of the Wisconsin Longitudinal Study participants,^8^ but was not found in Sweden.^7^

These historical associations for the cohort *in utero* in 1918–1919 are based solely on birth dates and cannot identify individuals whose mothers actually experienced infections. Thus, the analysis is restricted to population data on timing of infections and birth dates related to outcomes many decades after the pandemic. Thus we cannot readily distinguish direct effects on long-term health and well-being due to exposure to the infection itself versus the stress effects due to exposure to the pandemic environment. These socio-environmental stressors include potentially increased levels of uncertainty, undernutrition, under- and unemployment, poverty, and loss of loved ones. Despite this, the findings from the 1918 flu pandemic of lasting consequences for those exposed prenatally warrants study of similar programming effects from the current pandemic. Unlike the earlier pandemic, the current pandemic provides opportunity to study individuals with known *in utero* exposure to SARS-CoV-2 throughout their life based on known maternal infection and socio-environmental stresses. We can add further critical comparisons to individuals exposed *in utero* to the pandemic environment but with no maternal infection, which potentially allows disentanglement of the effects of exposure to infection versus pandemic environment.

### Long-term consequences of maternal and fetal exposure to viral infections

Maternal viral infections can affect the fetus or neonate through multiple pathways. Maternal infections can directly infect the fetus as well as the placenta.^9^ Viral species differ markedly in their impact on pregnant women and fetuses. For instance, HIV and hepatitis B virus are vertically transmitted in up to 40% of cases without the successful interventions that have almost eliminated direct transmission in the U.S. Fortunately, maternal transmission is rare for the coronaviruses MERS-CoV (2012) and SARS-CoV (2002)^10^ and the 2009 H1N1 influenza virus.^11^ And, no severe birth defects have been associated with prior coronaviruses, MERS-CoV (2012) and SARS-CoV (2002). In general, pregnancy increases virus-associated morbidity and mortality, as shown for influenza H1N1 in 2009 and SARS-CoV.^12^ ^,^ ^13^ These infections also caused more preterm delivery and low birth weight.^13^

Virus-related maternal inflammatory processes can impact the placenta and disturb *in utero* metabolism to cause intrauterine growth restriction (IUGR).^14^ Long-term consequences include impaired glucose and insulin metabolism and hyperactivity of the hypothalamic–pituitary–adrenal (HPA) axis.^15^ Historically, Barker and colleagues showed that IUGR and low birth weight which could be related to maternal infection or poor nutrition are associated with later life cardiovascular disease and obesity.^16^ In the Helsinki Birth Cohort (1934–1944), which suffered a suboptimal nutritional environment, smaller placentas predicted larger childhood body mass index (BMI) and increased adult cardiovascular disease.^17^ Maternal immune response to viruses and bacterial coinfections can impact fetal development with multi-generational behavioral and cognitive consequences.^18^

### Effects of COVID-19 on pregnant women and neonates

We summarize the main points of the COVID-19 literature published thus far, bearing in mind that these findings are preliminary as they are based on small numbers, specific locations, and, often, more severely ill patients.

#### COVID-19 in pregnant women

Pregnant women do not appear to be at increased risk of contracting SARS-CoV-2, as their rates of infection seem to parallel the rates of infection in their surrounding communities. When pregnant women do contract the SARS-CoV-2 virus, the majority experience a similar clinical course as age-matched non-pregnant women; however, reports increasingly show need for more hospitalization, mechanical ventilation, and intensive care.^19^ ^,^ ^20^ ^,^ ^21^ Thus far, the overall mortality rate from COVID-19 in pregnant women is not significantly different from non-pregnant women.^19^

As in the general population, a minority of pregnant patients develop severe and critical illness, with cardiopulmonary compromise, and even multi-organ failure. SARs-CoV-2 can cause an exaggerated inflammatory response and coagulation activation in some individuals, causing worse outcomes. Whether the immunologic changes of pregnancy affect the exaggerated inflammatory response is unknown. The limited data suggests that COVID-19 pregnancies have similar elevations of cytokines (IL-6, TNFα) and chemokines (CCL2, CXCL10) as other COVID-19 patients.^22^ Some postulate that the anti-inflammatory state of pregnancy required to prevent rejection of the fetus may actually help fight the inflammatory cascade caused by COVID-19.^23^ Severe COVID-19 disease is also associated with increased rates of thromboembolic events, despite prophylactic anticoagulation.^24^ Although pregnancy increases the risk of thromboembolic events, and COVID-19 could further increase thromboembolic events in pregnancy, this possibility has not been studied.

Little is known about the impact of the SARS-CoV-2 virus on the placenta and the impact of the associated inflammatory and thromboembolic responses on the placenta. There are some reports of neonatal infections from SARs-CoV-2 infection of the placenta and its transplacental transmission.^25^ The inflammatory and thromboembolic responses related to maternal COVID-19 disease may affect placental growth, vascular perfusion, and hypertensive disorders of pregnancy. One study of third trimester placentas from women infected with SARS-CoV-2 showed a significant increase in features of maternal vascular malperfusion, which may be related to the inflammatory and thromboembolic response.^26^ Importantly, these patients had moderate-to-severe COVID-19, but none were intubated. Together, the risks of these pathological markers was 3.3-fold above reference non-exposed population.

#### Birth outcomes

Rates of preterm birth may be increased by maternal SARS-CoV-2 infections, with estimates ranging from 12% to 27%.^27^ ^,^ ^28^ These may be overestimates because they over-represent the more severely ill pregnant women. The increased rates of preterm delivery may be related to fever and hypoxemia which increase risk for preterm labor, premature rupture of membranes, and abnormal fetal heart rates. However, asymptomatic but infected pregnant women also showed more preterm delivery than non-infected pregnant women. Estimates of low birth weight range from 12% to 20%.^20^ A meta-analysis reported 15.6% low birth weight which did not significantly differ by maternal COVID-19 status.^29^ Importantly, studies from Ireland and Denmark as well as anecdotal reports by doctors from several regions around the world suggest decreasing levels of preterm birth/birth of very low birthweight infants in the general population during the pandemic.^30^ ^,^ ^31^ This suggests that for some, the measures taken to control spread of the virus may have had some positive externalities.

Additionally, rates of C-section delivery are increased in pregnant women with SARS-CoV-2 infections. In a systematic review, C-sections occurred in 52%–85% of COVID-19 deliveries, which may also be overestimated.^27^ Early in the pandemic, C-section was the primary mode of delivery in some hospitals; given the lack of information about vertical transmission, there was concern that vaginal delivery might increase risk of infection in the neonate. C-section rates may also be increased due to abnormal fetal heart rate tracings related to maternal hypoxia and fever. Additionally, C-section may be chosen to relieve severe maternal disease, resulting in preterm birth.^32^

Stillbirths among women with COVID-19 so far have been rare and seem to have been related to very severe maternal illness. Infected mothers in one prospective study had significant several-fold increased rates of stillbirth.^33^ In another study, there was a general increase in stillbirths in the pandemic period in Britain.^34^

Data on effects of first and second trimester maternal infections is very limited, as is data for spontaneous abortions. There is a theoretical concern that fever in the first trimester may increase risk for miscarriage and congenital anomalies. Further studies of the outcomes of maternal infections, especially in the first and second trimesters are needed.

#### Neonatal outcomes

Most newborns born to mothers with COVID-19 disease have been healthy, and most commonly suffered from sequelae of prematurity rather than the effects of SARS-CoV-2. The rate of congenital infection from SARS-CoV-2 at this time is estimated at <3%, approximating the rates of other congenital infections.^35^ *In utero* transmission needs further study to substantiate reports of transmission, based on polymerase chain reaction assay for SARs-CoV-2 in the neonate and/or placenta, or elevated IgM antibodies.^25^ The few case reports of potential *in utero* transmission are complicated by the initial lack of criteria defining vertical transmission. Horizontal spread of the disease to neonates from family members or other household contacts occurs and is generally mild.^36^

### Ongoing/future studies

The COVID-19 cohort provides a natural experiment to examine the effects of maternal viral infection while infants were *in utero*. Such effects may span the lifecycle of these babies; during this time it may be possible to intervene to prevent later life problems. Cohort studies include the UCSF PRIORITY: Pregnancy Coronavirus Outcomes Registry and the American Academy of Pediatrics Society of Neonatal and Perinatal Medicine (AAP SoNPM) – National Perinatal COVID-19 (NPC-19) Registry, a collaboration of the AAP SoNPM with the Vermont Oxford Network (VON). Neither of these registries are currently supported for the long-term follow-up which is warranted by important scientific and clinical reasons.

We suggest that to capture the consequences of viral exposure *in utero* for childhood development and adult health COVID-19 birth cohort studies consider immediate collection of data from the mother, fetus, neonate, and placenta. These initial data should be followed by analysis of child growth and development and lifelong study of health, behavioral patterns, and cognitive functioning. Table 1 suggests multiple indicators for immediate measurement and measurement across the lifespan. Initial indications of disease presence, exposure, and severity are important including inflammatory cytokines in the mother and infant. Clinical data on the size and development of the infant both before and at birth and the placenta at birth should be routinely collected. Because relatively simple anthropometric measures routinely taken at birth serve only as a very crude proxy measure of the prenatal environment, we encourage studies using longitudinal ultrasound measurements of fetal development. Because hypocortisolemia was common in SARS-CoV-1, HPA axis activity should be studied in severe COVID-19 mothers (ChiCTR20000301150).^37^ Maternal genetics may also influence vulnerability to infection and impact on the fetus, shown for adult carriers of ApoE alleles.^38^ Molecular studies could include the placental transcriptome for genetic variants of gene expression (eQTLs) that predict childhood obesity and BMI.^39^ Epigenomic methylation of DNA and histones should be monitored across the lifespan of the infant. DNA methylation measures represented as epigenetic clocks that represent “aging” can be estimated in pediatric and then adult years.^40^ Socioeconomic status and educational status of the mother and child should also be assessed, as causes of adverse outcomes can be social as well as biological. Additionally, COVID-19 infection appears to be more common and severe in racial and ethnic minorities in the U.S.^41^ Moreover, preexisting conditions increase the severity of COVID-19 infection, which may have implications for transmission and neonatal outcomes. Our recommendations are oriented toward the United States as the situation may differ in low- and middle-income settings.

**Table 1.** Domains for evaluating effects of *in utero* exposure to COVID-19

Mother	Covid PCR test for infection, IgM and IgG antibodies; Characteristics of COVID disease if known; treatments received; other health conditions during pregnancy; DNA for identification of SNPs; Inflammatory markers (cytokines and chemokines); cortisol Socioeconomic and behavioral data on stresses, activities, and sleep during pregnancy		
Fetus and Neonate	Ultrasounds throughout pregnancy to track fetal development Covid PCR test for infection, IgM and IgG antibodies; Inflammatory markers (cytokines and chemokines) Birthweight, birth length, birth head circumference Gestational age DNA for identification of SNPs and epigenomics		
Placenta and membranes	Cord blood: Viral PCR and antibodies Placental weight and size; membrane and placental disc full thickness and maternal surface sections for histopathology, viral PCR and microbial culture, transcriptome, and eQTL		
	Infant/Child	Adult	Older Ages
Growth, Health, and Aging	Physical development (*WHO Child Growth Standards)*; epigenomics (e.g., pediatric DNA methylation clock);	Blood glucose and lipids; BMI; atherosclerosis and hypertension; immunity; DNA methylation clocks; biological age or pace of aging	Cardiovascular Disease, cancer, stroke, diabetes, mortality
Development, Behavior and Cognition	Neurodevelopment: Bayley Developmental Assessments; IQ scores; neuroimaging; educational achievement	Mental health, education, and occupation	Cognitive aging and neurodegenerative diseases

The child should be followed for indicators of growth, motor, and neurological development milestones. Throughout life, the standard blood-based indicators should be collected. Composite measures derived from clinical chemistry and physical exams can be used starting in childhood to categorize the “Pace of Aging.”^42^ Follow-up should include information on development of both pediatric (e.g., leukemia, asthma) and adult diseases (e.g., heart disease, cancer, stroke).

The COVID-19 pandemic also provides an opportunity to study the effects of *in utero* exposure to the pandemic environment and the effects of different regional and national policies to address viral spread. The COVID-19 pandemic has increased levels of stress, unemployment, food insecurity, and domestic violence, and diminished or disrupted prenatal care. These factors may have programming effects and/or direct effects on neonatal health. The pandemic-related lockdown policies may benefit some individuals; pregnant women staying home who may rest more, and have decreased exposure to other infections, and women with job stability, may benefit from less stress from work and commuting time. These benefits are evidenced by reports of decreased rates of preterm birth for the general population during the pandemic.

Studies would include mother and child dyads and a selection of mothers with symptomatic and nonsymptomatic COVID-19 diagnosis, as well as a group of mothers and babies with no *in utero* exposure. We suggest the desirability of enrolling an additional child born either shortly before or shortly after the COVID-19 pandemic to the same mother. These study groups would allow comparison between infants who were exposed to maternal infection and those who were prenatally exposed to the pandemic without having experienced maternal infection. These infants can also be compared to those who were born before or conceived after the pandemic, providing further insight on the effects of the pandemic environment. The inclusion of information on social and economic stresses will allow comparisons between countries taking different measures to reduce spread of the virus, These types of comparisons may give us further insights beyond the effects of COVID, such as socioeconomic and social policies that may decrease risk of preterm birth, which has eluded the maternal-infant health community for decades. This should be balanced by further investigating reports of generally increased stillbirth and potential contributors.

## Summary and conclusions

Maternal COVID-19 infections and exposure to the pandemic environment may have long-term effects on growth and aging for the cohort *in utero* during this pandemic based on the 1918 influenza birth cohort. From comparisons of the 1918 Influenza with the current COVID-19 pandemic, we suggest studies of markers from birth through adulthood that are indicators of the altered development and accelerated aging that could be experienced in the century ahead.

We need to evaluate whether the effects of COVID-19 can be generalized to other viruses. While the current pandemic is similar in some ways to the earlier, there are important differences. COVID-19 resembles the 1918 Flu in that many infections were relatively uncomplicated cases, but deadly for a small proportion of cases. Surprisingly, given the 100 year difference in time, the mortality during the two pandemics appears fairly similar in New York at the height of the pandemic; an increase over baseline of about 200 deaths per 100,000 population in the 1918 epidemic and an increase of about 150 per 100,000 in 2020.^43^ A key difference between the 1918 pandemic and the current COVID-19 pandemic is the more significant role of secondary bacterial infections, in comparison to the current severe lung disease, multi-organ damage, inflammatory response, and thromboembolic tendency reported for the most serious cases of COVID-19. Secondary bacterial infections related to COVID-19 have mostly been effectively managed with antibiotics. Additionally, the 1918 flu was particularly deadly for people aged 20 to 40, or those in the childbearing years; in contrast, the SARS-CoV-2 virus causes higher mortality among older persons. While the long-term outcomes may not be the same as 1918, it is important to study the effects of SARS-CoV-2 during pregnancy in terms of consequences for later life health and aging.
